# Patient Satisfaction with Resin Infiltration Treatment for Masking Noncavitated White Spot Lesions on Anterior Maxillary Teeth: Two Case Reports

**DOI:** 10.1155/2022/9180553

**Published:** 2022-09-09

**Authors:** Rana A. Alamoudi, Asmaa A. Yaseen, Amjad S. Alharthi, Sara M. Bagher

**Affiliations:** ^1^Pediatric Dentistry Department, Faculty of Dentistry, King Abdulaziz University, Jeddah 21589, Saudi Arabia; ^2^Pediatric Dentistry Resident, Department of Pediatric Dentistry, King Salman Armed Forces Hospital, Tabuk, Saudi Arabia; ^3^Pediatric Dentistry Resident, Department of Pediatric Dentistry, King Abdulaziz University, Jeddah, Saudi Arabia

## Abstract

**Aim:**

We assessed patient satisfaction with resin infiltration treatment outcomes for masking postorthodontic treatment noncavitated white spot lesions on anterior maxillary teeth.

**Background:**

White spot lesions (WSLs) are opaque white areas of demineralization. The lesion may remineralize over time, but the opaque color usually remains, retaining the undesirable tooth color. *Case Description*: Resin infiltration (RI) was administered to two patients with a total of 10 anterior maxillary teeth diagnosed with noncavitated WSLs. Immediately after treatment, patients were asked about their comfort during the RI treatment and their level of satisfaction with treatment outcomes. Two weeks post-treatment, the two patients were asked again about their level of treatment outcome satisfaction and if they thought they needed a second treatment. Both reported that they were comfortable during the treatment and were extremely satisfied with the achieved treatment outcomes immediately and two weeks after the treatment and did not feel that they would need to seek further treatment to reach the desired outcome.

**Conclusion:**

Resin infiltration is a comfortable, conservative treatment option providing satisfactory outcomes in masking noncavitated WSLs on anterior maxillary teeth after orthodontic treatment. *Clinical Significance*. Resin infiltration is a conservative treatment option to mask noncavitated WSLs after orthodontic treatment.

## 1. Introduction

Dental caries results from an imbalance between demineralization and remineralization processes after the intake of fermentable carbohydrates [1]. As the microorganisms within the dental plaque biofilm are exposed to fermentable carbohydrates and sugars, an organic acid is produced [2]. This acid dissolves the intercrystalline spaces within the enamel [3], which creates a pathway for the transport of minerals out of the enamel [4].

Caries in enamel is first observed clinically as a noncavitated incipient white spot enamel lesions (WSLs). The surface zone of the WSLs acts as a barrier to bacterial invasion and remains highly mineralized, while the lesion's body loses its minerals [5] and the pores become filled with water or air [6]. Therefore, WSLs appear clinically as opaque white areas of demineralization [7].

Orthodontic fixed appliance increases the likelihood of unsatisfactory oral hygiene, leading to dental plaque adherence to the appliance surfaces. They also limit the ability of saliva, lips, tongue, and cheeks to self-cleanse, which increases the risk of WSL development [8, 9]. Poor oral hygiene, increased the treatment duration, and lack of fluoride supplement use during the orthodontic treatment are risk factors identified to increase the risk of WSL development [10].

Therefore, patient education, oral hygiene instruction, and patient compliance are the most significant prophylactic measures against the formation of WSLs [11]. Professional prophylactic cleaning and routine use of fluoride-containing mouthwash have also been recommended to prevent WSLs formation during fixed orthodontic treatment [12].

The treatment options of post-orthodontic treatment WSLs range from very conservative (natural remineralization) to more invasive (tooth preparation and restoration) procedures, based on the severity of the lesion [13]. Conservative methods for remineralization usually include proper oral hygiene measures, which requires patient's compliance and involves multiple applications of fluorides, and/or casein phosphopeptide-amorphous calcium phosphate containing products [14, 15]. Recently, a few studies reported that the use of biomimetic hydroxyapatite containing toothpaste forms a coating on the enamel surface, promotes superficial enamel repair, and aids in preventing secondary caries around restorations [16, 17, 18].

More invasive treatment modalities include bleaching, microabrasion, resin infiltration (RI), resin restoration, and indirect restoration [19]. Gentle tooth bleaching for WSLs together with the incorporation of nano-bioactive glass ionomer, nano-hydroxyapatite, and nano-amorphous calcium phosphate into the bleaching agents can reduce the negative side-effects associated with bleaching [20]. Microabrasion is a more invasive procedure that bleaching for the treatment of superficial WSLs and includes both chemical and mechanical processing of the enamel surface [6]. Finally, RI is a minimally invasive treatment modality that involves the utilization of strong acid that opens the enamel tubules, which permits a low-viscosity resin to penetrate and replace the air or water in the pores and improves the refractive index of the affected enamel to resemble normal enamel, enhancing lesion appearance immediately [21, 22].

Multiple studies evaluated the esthetic effect of RI in managing postorthodontic treatment WSLs and reported that RI treatments is very effective in camouflaging postorthodontic treatment WSLs immediately after application [23–26]. But only one study evaluated patients' satisfaction with the RI treatment outcomes [25]. Therefore, the purpose of this report was to assess patient satisfaction with resin infiltration treatment outcomes for masking postorthodontic treatment noncavitated white spot lesions on anterior maxillary teeth.

## 2. Case Presentation

The two patients were referred by the Orthodontic Department at King Abdulaziz University Faculty of Dentistry (KAUFD) after completing their orthodontic treatment with the chief complaint of WSLs in the labial surfaces of their upper anterior teeth. At KAUFD, patients receiving orthodontic treatments are always encouraged to maintain proper oral hygiene instructions and to use fluoride-containing toothpaste and mouthwash during their treatment and after the debonding. The dental records of patients who finished their orthodontic treatment within the last two weeks were reviewed. Five healthy patients diagnosed with postorthodontic treatment WSLs were identified and contacted by phone. Three of them answered the phone and agreed to attend a scheduled appointment to discuss the treatment options for their post-orthodontic treatment WSLs. Two of them were interested in the RI treatment for their postorthodontic treatment WSLs and were examined clinically by a trained dentist using the International Caries Detection and Assessment (ICDAS) method, which was created to differentiate between the different stages of carious lesions (pit and fissures, and smooth surface) using visual and tactile techniques [28]. For smooth surface lesions, the ICDAS score was 1 if the enamel surface of the anterior maxillary teeth appeared sound, but a white or brown lesion was visible after prolonged air drying. If there was a distinct visual change in the enamel and the lesion appeared white or brown, the ICDAS score was 2. Only WSLs with ICDAS scores of 1 or 2 received RI treatment. Fluorosis was not detected (using Dean's fluorosis index), nor was amelogenesis imperfecta (according to the Witkop classification) [29] on any anterior maxillary teeth of the two patients.

The first patient was a 16-year-old female with four teeth (maxillary central and lateral incisors) diagnosed clinically with noncavitated WSLs (ICDAS scores of 2), and the second was a 23-year-old male with six teeth (maxillary central incisors, lateral incisors, and canines) diagnosed clinically with noncavitated WSLs (ICDAS of 2). Both patients finished their orthodontic treatment three months prior to RI application. They were nonsmokers and reported brushing and flossing their teeth at least once daily during their orthodontic treatment. Neither patient was diagnosed with WSLs determined to have ICDAS scores >2 on their anterior maxillary teeth.

Before the treatment, the patients were asked to report on a numeric scale of 1 to 5, the extent to which they were uncomfortable or annoyed by the WSLs on their anterior maxillary teeth that appeared after orthodontic treatment. A score of 1 was defined as being comfortable and not annoyed at all, and 5 represented extremely uncomfortable and annoyed. Both patients reported feeling extremely annoyed (score = 5) by the WSLs on their anterior teeth.

**Figure 1:**
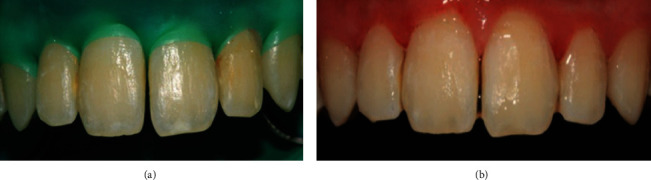
(a) Case 1, pretreatment. (b) Post-treatment with resin infiltration.

**Figure 2:**
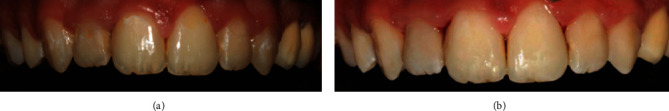
(a) Case 2, pretreatment. (b) Post-treatment with resin infiltration.

The noncavitated WSLs were treated with RI (Icon, DMG America; Englewood, NJ, USA) by the same dentist according to the following protocol: (1) the facial surfaces of the affected teeth were cleaned with a rubber cup and plain pumice, then rinsed and dried for 30 seconds; (2) teeth were isolated with a rubber dam, and then 15% hydrochloric acid gel (Icon Etch) was placed on the affected teeth for two minutes, rinsed with water, and air-dried for 30 seconds; (3) ethanol (Icon Dry) was brushed on and dried for 30 seconds; (4) teeth were then visually inspected to determine whether the whitish opaque discoloration lesions had diminished significantly and if the discoloration was not concealed, the previous steps were repeated; (5) once the desired result was achieved, RI was applied to the teeth surfaces for three minutes, and cotton roll was used to remove excess resin material. The teeth were flossed and light-cured for 40 seconds according to the manufacturer's instructions. A second layer of resin was applied for one minute following the same previous steps. Finally, the teeth surfaces were polished using a spiral polishing wheel. The pre- and post-RI treatment outcomes of the first and second cases are shown in Figures 1 and 2, respectively.

Immediately after the RI procedure, both patients were asked whether they felt comfortable during the treatment, their level of satisfaction with the treatment outcomes and if they would recommend this treatment to others. Both reported that they were comfortable during the treatment and extremely satisfied with the achieved treatment outcomes. Moreover, both reported that they would recommend this treatment to others.

Two weeks post-treatment, the patients were contacted by phone and asked again about their level of satisfaction with the treatment outcomes and if they were considering a second treatment visit. Both were extremely satisfied with the achieved treatment outcomes and reported that the outcomes had improved since immediately after the treatment. Furthermore, they did not feel they would need to seek further treatment to reach the desired outcomes.

## 3. Discussion

High patient satisfaction with RI treatment outcomes in masking noncavitated WSLs on anterior maxillary teeth after orthodontic treatment was reported by both presented cases. WSLs are considered one of the major side effects of orthodontic treatment [30, 31], especially in maxillary lateral incisor, maxillary canine, and central incisors, respectively [32].

A study conducted by Kim et al. [33] to evaluate clinically the effectiveness of RI in masking WSLs after orthodontic treatment. The time lapse between the orthodontic bracket debonding and RI treatment for the included WSLs was 21 months. They reported immediately after the RI application; most of the lesions were completely masked but others were only partially masked or unchanged [33]. This was explained by the fact that the esthetic effect of RI is more effective in active and shallow lesions compared to deeper and inactive lesions with thinker outer protective layer. Therefore, it was recommended to do the RI treatment as soon as possible after bracket removal to avoid lesion progression and the loss of the surface integrity [6, 33]. In our cases the RI treatment was provided a few weeks after the removal of the orthodontic brackets while the lesions were still active and shallow.

The esthetic effect of RI in managing postorthodontic WSLs was reported to be stable 2 [23], 6 [24], and 12 months after the treatment [25, 26]. In the presented cases, patient's satisfaction with the treatment outcomes was done immediately after the treatment and two weeks later. Further follow-up for a longer duration is recommended to ensure that the level of patients' satisfaction with the treatment outcomes is maintained.

A recent study by Solinas et al. [17] reported that the application of biomimetic nanohydroxyapatite for the treatment of incisor and molar hypomineralization for six months in a 4-year-old patient resulted in a significant decrease in the hypersensitivity and improved the enamel appearance for the maxillary incisors. Further studies to evaluate the effect of biomimetic nanohydroxyapatite are recommended in hope that more invasive treatment options can be avoided.

Hughes and Roseberg [27] evaluated patient satisfaction with the esthetic changes resulting from RI treatment of WSLs associated with orthodontic treatment. Before the treatment, patients reported that their smile was extremely important, and that WSLs bothered them. Immediately after treatment, patients were satisfied with the achieved outcomes [27], consistent with our two case reports.

It was also previously reported that combining home bleaching and RI for treating teeth in different stages of fluorosis improves patient confidence and satisfaction with their appearance [34]. Another recent *clinical study showed that patients were highly satisfied* with the RI procedure duration for the treatment of fluorosis lesions [35].

This report demonstrates that RI can be considered an effective and reliable treatment option for masking noncavitated WSLs due to its deep penetration. We recommend that future studies include longer follow-up periods examining the long-term success and relapse rate of RI as a conservative treatment option for noncavitated WSLs.

## 4. Conclusions

Resin infiltration is a comfortable, conservative treatment option that provides satisfactory outcomes in masking noncavitated WSLs on anterior maxillary teeth after orthodontic treatment.

## Data Availability

The data used to support the findings of this study are available from the corresponding author upon request.

## Additional Points

Resin infiltration is an effective, conservative treatment option to mask noncavitated WSLs after orthodontic treatment.
